# Tackling Post-COVID-19 Rehabilitation Challenges: A Pilot Clinical Trial Investigating the Role of Robotic-Assisted Hand Rehabilitation

**DOI:** 10.3390/jcm13061543

**Published:** 2024-03-07

**Authors:** Ana Cisnal, Gonzalo Alonso-Linaje, Juan Carlos Fraile, Javier Pérez-Turiel, Pablo Álvarez, Socorro Martinez

**Affiliations:** 1 Instituto de las Tecnologías Avanzadas de la Producción (ITAP), University of Valladolid, Prado de la Magdalena 3-5, 47011 Valladolid, Spain; gonzalo.alonso.alonso-linaje@uva.es (G.A.-L.); jcfraile@uva.es (J.C.F.); jpturiel@uva.es (J.P.-T.); 2 Centro Hospitalario Padre Benito Menni, P° Juan Carlos I, 10, 47008 Valladolid, Spain; pavian.valladolid@hospitalarias.es (P.Á.); smartinez.valladolid@hospitalarias.es (S.M.)

**Keywords:** case report, COVID-19, musculoskeletal sequalae, physical rehabilitation, robotic therapy

## Abstract

**Background:** Prolonged hospitalization in severe COVID-19 cases can lead to substantial muscle loss and functional deterioration. While rehabilitation is essential, conventional approaches face capacity challenges. Therefore, evaluating the effectiveness of robotic-assisted rehabilitation for patients with post-COVID-19 fatigue syndrome to enhance both motor function and overall recovery holds paramount significance. Our objective is to assess the effectiveness of rehabilitation in post-COVID-19 patients with upper extremity impairment through the utilization of a hand exoskeleton-based robotic system. **Methods:** A total of 13 participants experiencing acute or limited functional or strength impairment in an upper extremity due to COVID-19 were enrolled in the study. A structured intervention consisted of 45 min therapy sessions, conducted four times per week over a six-week period, utilizing a hand exoskeleton. The research employed standardized health assessments, motion analysis, and semi-structured interviews for pre-intervention and follow-up evaluations. Paired sample *t*-tests were employed to statistically analyze the outcomes. **Results:** The outcomes showed a reduction in overall dependence levels across participants, positive changes in various quality of life-related measurements, and an average increase of 60.4 ± 25.7% and 28.7 ± 11.2% for passive and active flexion, respectively. **Conclusions:** Our data suggest that hand exoskeleton-based robotic systems hold promise to optimize the rehabilitation outcomes following severe COVID-19. Trial registration: ID NCT06137716 at ClinicalTrials.gov.

## 1. Introduction

Coronavirus disease 2019 (COVID-19) is an infectious illness caused by the severe acute respiratory syndrome coronavirus 2 (SARS-CoV-2). On 11 March 2020, the World Health Organization (WHO) declared COVID-19 a global pandemic. By early November 2020, there were nearly 50,000,000 reported cases of COVID-19 and over 1,250,000 deaths worldwide [1]. As of the end of January 2024, the cumulative cases of COVID-19 reached 774,469,939 globally, with 7,026,465 deaths, according to data compiled from the Johns Hopkins University (JHU) Coronavirus Resource Center’s COVID-19 Map, which concluded on 7 March 2023, and the WHO’s COVID-19 Dashboard, covering data from 7 March 2023, onward [2].

However, it is imperative to acknowledge the inherent challenges in accurately reporting COVID-19 cases and mortality rates on a global scale due to significant disparities in testing infrastructure, diagnostic methodologies, and the certification process. Moreover, the profound impact of the pandemic extends beyond the directly attributable deaths, resulting in widespread collateral damage to both lives and livelihoods. Notably, a comprehensive study examining excess deaths in 2020 and 2021 revealed a staggering total of 14.83 million excess deaths worldwide, far exceeding the reported COVID-19 death toll of 5.42 million during the same period [3].

The COVID-19 pandemic has had profound implications on global health, with a multitude of consequences affecting individuals who have contracted the virus. Moreover, the COVID-19 pandemic placed immense pressure on healthcare systems worldwide, particularly in intensive care units (ICUs). COVID-19 patients are at risk of developing post-intensive care syndrome, resulting in a decline in physical functional status [4].

Among the severe cases, patients requiring ICU admission due to COVID-19 often face a lengthy and challenging recovery process. Common symptoms include fatigue, dyspnea (shortness of breath), post-traumatic stress disorder (PTSD), depression, concentration problems, pain, voice changes, cough, memory issues, continence problems, and dysphagia [5]. While the primary focus has been on respiratory complications and the impact on vital organs, there is a growing recognition of the significant motor body impairments experienced by individuals who have spent an extended period in the ICU [6]. These impairments, which hinder their personal care and performance of daily activities, can arise from a combination of factors, including the direct effects of the virus, prolonged immobilization, and the utilization of various life-saving medical interventions [7].

Numerous studies have analyzed the reduced physical capacity of post-COVID-19 patients discharged home after acute and post-acute care hospitalization, often resulting in severe disability. Research has identified a relationship between the loss of muscle mass and the duration of hospitalization [8]. Prolonged immobilization during extended hospitalization, particularly in severe COVID-19 cases, leads to muscle mass and function decline. In fact, studies have shown that peripheral muscle strength decreases by about 20% per week of bed rest in hospitals [9,10]. This muscle loss is most pronounced in the initial 2–3 weeks of immobility. Atrophy induced by severe COVID-19 hospitalization can result in persistent muscular dysfunction for several months [11]. Long-term consequences include severe muscle weakness, extreme fatigue, reduced mobility, diminished activities of daily living (ADLs), and neuro-psychological issues, especially in ICU-admitted COVID-19 patients [12].

A study involving 100 post-COVID-19 patients reported that 21% of ICU-admitted patients experienced worsened mobility, and 16% of non-ICU COVID-19 cases also experienced a decline in mobility [5]. Epidemiological data also indicate disabling consequences due to COVID-19′s impact on the central and peripheral nervous systems, whether through viral migration to the brain (resulting in hypogeusia and hyposmia) or as an adverse effect of the respiratory syndrome and ICU stay (such as post-intensive care syndrome and hypoxic encephalopathy with persistent executive dysfunction). A review of 32 studies reported the incidence of new neurological events ranging from 6% to 67% of hospitalized COVID-19 patients, with over half of the patients exhibiting neuromuscular impairments [13].

Rehabilitation has been proven crucial for post-COVID-19 patient recovery, addressing fatigue and improving functional status even in chronic stages [14]. Thus, it is essential to provide appropriate respiratory and neuromotor rehabilitation plans for COVID-19 patients to restore their previous functional status [4,13]. However, considering the significant number of post-COVID-19 patients requiring intensive care, the demand for traditional physical rehabilitation exceeded hospital capacities. Expanding healthcare personnel to meet these rehabilitation needs would lead to excessive public spending. An alternative approach is the utilization of robotic systems, which enable patients to rehabilitate autonomously. These systems allow multiple patients to undergo rehabilitation simultaneously, supervised by a single healthcare professional.

Numerous studies have demonstrated the effectiveness of robotic-assisted rehabilitation for patients with reduced mobility due to other diseases [15,16,17,18,19]. In addition to cost reduction, it offers advantages over traditional methods, including independent rehabilitation, increased time for patient rehabilitation time, and motivation [20,21]. Robots also enable simple and objective measurements to evaluate the patient’s motor recovery [22] and provide personalized rehabilitation tailored to the patient’s specific needs [23]. Finally, robotic-assisted rehabilitation integrates virtual reality-based video games, which have shown increased effectiveness by enhancing patient motivation [24].

This manuscript presents the findings of a clinical trial that explores the efficacy of hand rehabilitation utilizing an exoskeleton-type robot among COVID-19 patients. Notably, there is a paucity of studies investigating this specific topic [25]. To our knowledge, only three studies have examined the effectiveness of robotic rehabilitation therapies in COVID-19 patients. These studies targeted lower limb robotic rehabilitation using LOKOMAT (Hocoma, Volketswil, Switzerland) [26], ANDAGO (Hocoma, Volketswil, Switzerland) [27], and LUNA EMG (EGZOTech, Gliwice, Poland) [28], thereby neglecting hand rehabilitation as a specific focus. Thus, our research aims to fill this knowledge gap by offering novel insights into the effectiveness of exoskeleton-based hand-assisted rehabilitation for COVID-19 patients and its potential impact on their quality of life. This contribution holds paramount importance in advancing our understanding of the potential benefits of such interventions, particularly given the limited existing research in this domain.

## 2. Materials and Methods

### 2.1. Robotics Hand Exoskeleton

In this study, the RobHand rehabilitation platform was used to evaluate motor function enhancement through robotic-assisted hand therapies. The RobHand exoskeleton employs a direct-driven under-actuated serial four-bar linkage mechanism, utilizing five L12-30-100-6-I linear actuators (Actuonix Motion Devices Inc., Saanichton, BC, Canada), each with a 30 mm stroke length and a maximum force of 23 N. This under-actuated design allows for the direct control of the metacarpophalangeal (MCP) joints and indirect control of the proximal interphalangeal (PIP) joints, providing a considerable range of motion for the MCP joint (72° flexion and 2° extension) [29].

**Figure 1:**
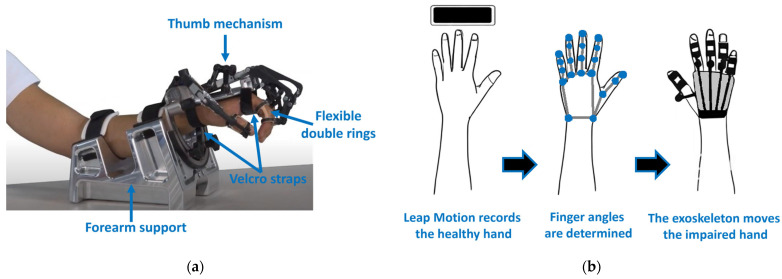
Overview of the RobHand system: (**a**) The exoskeleton is secured to the hand through flexible double rings and Velcro straps, with the thumb correctly positioned using the designated mechanism. The exoskeleton is affixed to the forearm support; (**b**) Illustration depicting the operation of bilateral therapies based on the Leap Motion. The Leap Motion provides directional vectors of the hand’s digits, facilitating the determination of finger angles to precisely control the movement of the hand exoskeleton.

Attachment to the user’s hand is facilitated by Velcro straps on the palmar side and flexible double rings made of Filaflex 82a material (Recreus Industries, S.L., Alicante, Spain). This design ensures adaptability to different finger sizes, securing the exoskeleton to each finger. A multi-articulated passive holder mechanism accommodates diverse thumb dimensions. The integrated forearm support mitigates forces and torques, enhancing patient comfort during rehabilitation (Figure 1a). The design prioritizes ergonomics and user adaptability, reflected in a high user satisfaction score (4 out of 5) on the Quebec User Evaluation of Satisfaction with Assistive Technology 2.0 Scale (QUEST 2.0) [30].

The exoskeleton facilitates both passive and bilateral therapies. In passive therapies, therapists set the range of flexion and extension for each finger, and the exoskeleton performs repetitive movements within this range [31]. On the other hand, bilateral therapies involve replicating the movements of the healthy hand onto the hand exoskeleton worn on the affected hand [32]. These bilateral therapies are based on Leap Motion Controller (Ultraleap., San Francisco, CA, USA). During bilateral training, the Leap Motion captures directional vectors of the proximal phalanges and metacarpals for each finger. By identifying these vectors and applying the scalar product formula, the angle between these two vectors (the MCP angle) is determined for each detected phalanx within the Leap Motion’s field of view. These angles are transmitted as control signals to precisely adjust the movements of the MCP joint angles of the exoskeleton, aligning with the unimpaired hand movement (Figure 1b).

### 2.2. Recruitment

Participants were recruited at the Centro Hospitalario Benito Menni (Valladolid, Spain) by a therapist referral or through a participant database. All participants gave written informed consent to participate in the study. All experimental procedures were approved by the Medicine Research Ethics Committee of the Hospital Clínico Universitario de Valladolid (CASVE-NM-22-575). Individuals over the age of 30 years who were admitted to Centro Hospitalario Benito Menni for COVID-19 infection and had acute or limited functional or strength impairment in at least one of the upper extremities were eligible to participate in the study. Individuals were confirmed to have COVID-19 through RT-PCR (Reverse Transcription Polymerase Chain Reaction) testing, which is considered the gold standard for diagnosing active infection with the SARS-CoV-2 virus. The presence of behavioral disorders, dementia (loss of memory of cognitive functions), disorders of consciousness (confusional states and drowsiness), uncontrolled or severely limiting delusions and hallucinations, infectious skin diseases, a risk of epileptic seizures, severe visual impairments, severe spasticity with a Modified Ashworth Scale > 2, joint stiffness in the wrist and fingers, and pain with a score > 8 on the Visual Analog Scale (VAS) during the mobilization of the affected hand were defined as the exclusion criteria.

### 2.3. Study Protocol and Setup

The study was conducted from July 2022 to February 2023 at the occupational therapy area of the Centro Hospitalario Benito Menni (Valladolid, Spain). Over six consecutive weeks, patients underwent a robotic-based hand therapy program using the RobHand platform. The therapy sessions, lasting 45 min each, were conducted four times per week resulting in 24 sessions. Within each session, a total of six exercises, consisting of three passive exercises and three bilateral exercises, were performed in an alternating manner. The exercises were executed in the following sequence: (1) Bilateral hand opening and closing; (2) Passive independent finger opening and closing; (3) Bilateral pinch; (4) Passive hand opening and closing; (5) Bilateral hand opening and closing; (6) Passive pinch.

**Figure 2:**
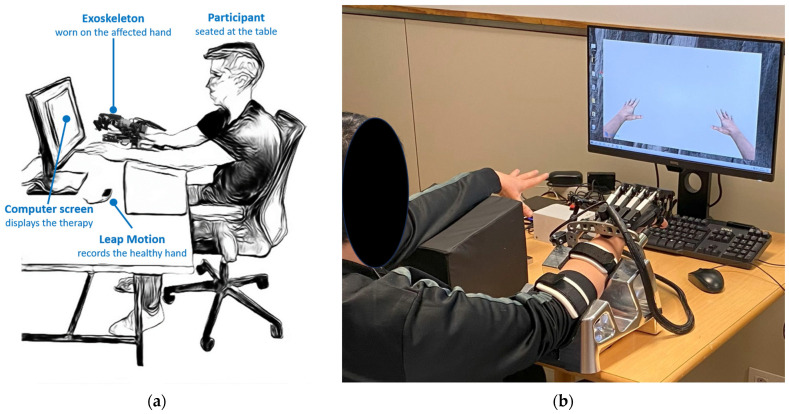
Study setup: (**a**) The participant was seated at the table in front of the computer screen. The participant was wearing the hand exoskeleton on the affected hand, while the Leap Motion recorded the healthy hand; (**b**) A patient with right-side impairment engaged in bilateral hand-opening and -closing exercises.

At the beginning of each session, participants were seated at an adjustable-height table in front of the computer screen to ensure maximal comfort. The hand exoskeleton was then worn on the affected hand of the participants, with a study coordinator in charge of the placement procedure. The exoskeleton’s base plate was affixed to the hand using Velcro straps and was secured to the forearm support. Subsequently, flexible rings were attached, and the thumb’s position was manually adjusted. Once the exoskeleton was in place, the unaffected forearm was positioned on a support, aligning both hands at the same height. Additionally, the unaffected hand was positioned within the field of view of the Leap Motion device, which was situated on the table (Figure 2). Prior to commencing the therapy session, the study coordinator adjusted the maximum angles for flexion and extension of each finger according to the motor capabilities of the respective patient.

### 2.4. Outcome Measures

The clinical trial utilized a mixed-method approach incorporating clinical evaluation, motion analysis, standardized health assessment, and semi-structured interviews. Patient interviews, motion analysis, and clinical evaluation instruments were used to assess the domains of the International Classification of Functioning, Disability and Health (ICF), as shown in Table 1. Clinical evaluation instruments used included the Functional Independence Measure (FIM) instrument, the SF-36 Health Questionnaire, and the Barthel Index (BI).

The Barthel Index (BI) modified by Granger et al. [33] is an ordinal scale for measuring the ability of an individual to independently execute 15 activities of daily living (ADL) related to mobility and self-care. The BI aims to evaluate the degree of independence, with a final score ranging from 0 to 100, where 0 indicates full dependence.

The Functional Independence Measure (FIM) [34] was designed to provide an indicator of disability independent of a patient’s impairment. The scale consists of 18 items to evaluate functional abilities in the area of communication, locomotion, self-care, social cognition, sphincter control, and transfers. Each of these items is quantified utilizing a 7-point Likert scale. The total score, obtained by summing the individual item scores, spans from 18 to 126, with 18 denoting a state of complete dependence.

The SF-36 Health Questionnaire [35] consists of 36 items designed to assess health-related quality of life. It encompasses eight scales, each comprising 2 to 10 items, which aim to evaluate various health attributes. These scales include physical functioning (PF), role-physical (RF), bodily pain (BP), general health (GH), vitality (VT), social functioning (SF), role-emotional (RE), and mental health (MH).

The range of motion was analyzed by goniometry measurements using the HandTutor glove (Meditouch Ltd., Tnuvot, Israel). This rehabilitation device facilitates repetitive and intensive active finger and wrist movements. Sensors located on the front and back of the glove monitor finger and wrist movements, providing various evaluation measurements. Among the available metrics, this study only utilized and analyzed the passive and active extension of the fingers. Although the sensors are highly sensitive to small variations in the patients’ movements, measurements may vary depending on glove positioning [36]. Hence, three measurements of passive and active flexion were taken and averaged at both the initial and follow-up evaluation.

Additionally, a semi-structured interview was created to provide additional information about personal and environmental factors and participation. Questions included in the structured questionnaire were as follows: (1) What are your current living arrangements (alone, with family or significant other, or in a residence of hospital facility)? (2) What is your level of education? (3) What is your level of use of the following electronic devices: mobile phone, gaming console, and computer?

Two interviewers were present for all interviews and initial and follow-up clinical evaluations with participants. Interviewers were trained and had experience in the administration of questionnaires. One interviewer administered the questions to the participant, and the other observed. Both interviewers transcribed answers to these questions verbatim. Both interviewers reviewed the transcriptions for accuracy, and changes were made if needed. Answers were entered into a spreadsheet and de-identified for later statistical analysis. Patient condition was evaluated using clinical evaluation instruments and motion analysis at baseline (3 days before the first training session) and at endpoint (3 days after the last training session). The same examiner (P.A.) evaluated all participants.

### 2.5. Statistical Analysis

The study outcomes were reported as mean ± standard deviation, with the ranging specified as minimum to maximum. In order to evaluate potential enhancements in functional outcomes following the rehabilitation program, the analysis employed paired-sample *t*-tests to assess alterations in motion analysis and clinical evaluation scores between the initial and follow-up assessments. A significance threshold of 0.05 was rigorously adhered to throughout the statistical analysis process. All data analysis was conducted using the R statistical software (Vienna, Austria, Version number 4.2.2).

## 3. Results

### 3.1. Participants

**Figure 3:**
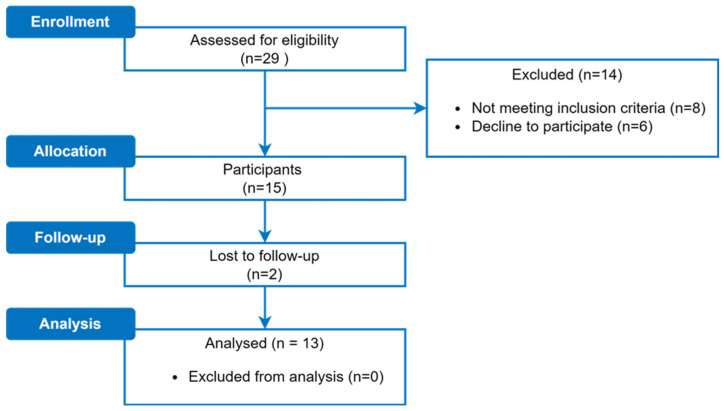
Participant flow diagram.

A total of 29 participants were recruited from medical records at the Hospital Benito Menni. Of this initial cohort, 8 were excluded after further review and 6 declined to participate due to a lack of time or interest, resulting in 15 participants who entered the study and completed personal interviews and initial assessments. However, two of them did not complete the follow-up evaluations due to unrelated clinical complications. Thus, a total of 13 participants successfully completed the study and were included in the analysis (Figure 3).

Table 2 provides an overview of the demographic characteristics, clinical information, living situation, educational background, and use of electronic devices in the study cohort. The cohort comprised thirteen subjects, including eight males and five females, with a mean age of 60.2 ± 16.0 years old [range 41–83]. All had experienced upper limb impairment (five with left impairment and eight with right impairment) as a consequence of COVID-19 infection and completed the study. The participants were infected on average 3.8 ± 2.1 months prior to the study [range 1–8.5]. Their hospitalization duration averaged 52.2 ± 40.1 days [range 10–109], with a ward stay of 38.7 ± 27.0 [range 10–78]. Among the 13 patients, five of them required ICU admission, with a total duration of 27.0 ± 11.2 days [range 7–33]. Additionally, five patients had suffered a stroke 2.8 ± 2.3 months [range 1–7] before the study, and one of them was also diagnosed with lymphoma one year before.

Three patients were housed in residential or hospital facilities, whereas ten patients resided in their own homes. Among these ten, eight cohabitated with a family member or their partner, and two of them live independently.

Concerning the participants’ educational background, four individuals hold university qualifications, including two engineers, one medical doctor, and one individual with an unknown degree. Additionally, four participants have successfully completed primary education, one has achieved secondary education, and two have earned high school diplomas. Furthermore, two individuals have opted for alternative educational pathways, specifically in the realm of professional training.

Regarding the use of electronic devices, which involves fine hand motor function, four participants rarely use mobile phones, two use them occasionally, and seven use them frequently. When it comes to video game consoles, eleven participants never use them, while two use them occasionally. As for computers, seven participants never use them, one uses them occasionally, and five use them frequently.

### 3.2. Outcomes Measures

As a general overview of the health status of the participants, the level of independence based on the Barthel Index is presented in Table 3. In the pre-intervention assessment, it was observed that two participants exhibited total dependence, six displayed severe dependence, four demonstrated moderate dependence, and one slight dependence. Following the intervention program, significant improvements were noted, with one individual remaining in the category of total dependence, one in severe dependence, five in the category of moderate dependence, one in slight dependence and, encouragingly, five participants achieving a state of complete independence.

**Figure 4:**
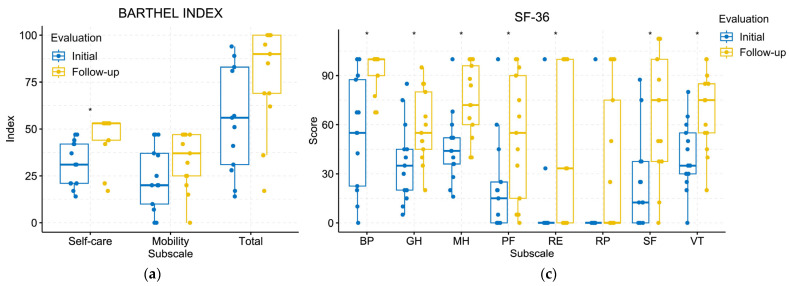

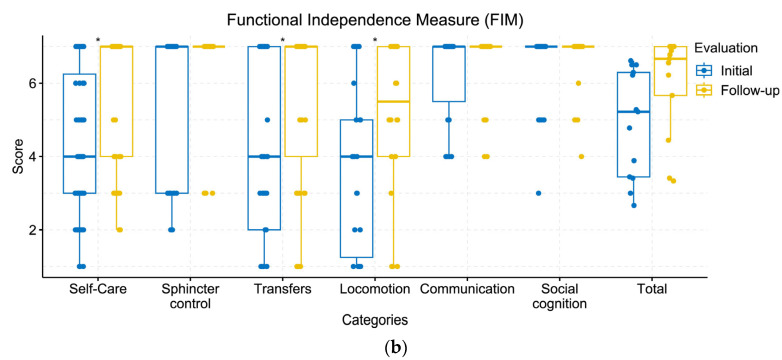
Results of the health and functional assessment questionaries administrated at the initial and follow-up evaluations for the 13 participants: (**a**) Results of the Barthel Index (BI); (**b**) Results of the Functional Independence Measure (FIM); (**c**) Results of the SF-36 questionnaire. * indicates significant differences identified by a paired *t*-test at the *p* < 0.05 level.

The results obtained from the assessments using the BI, FIM, and SF-36 instruments at the initial and follow-up evaluations are depicted in Figure 4. For the BI (Figure 4a), no statistically significant differences were observed in the overall score (t = −2.0505; *p*-value = 0.0514). Although a significant improvement was noted in the self-care sub-index (t = −2.8295; *p*-value = 0.0093), no such improvement was observed in the mobility sub-index (t = −1.3841; *p*-value = 0.1793). Concerning the FIM (Figure 4b), no significant differences were found in the total score (t = −1.9450; *p*-value = 0.0636). However, a detailed examination of individual items revealed a significant improvement in several aspects, including self-care (t = −5.5095; *p*-value < 0.0001), transfers (t = −2.8613; *p*-value = 0.0054), and locomotion (t = −2.2095; *p*-value = 0.0318). No statistically significant differences were observed in other items: sphincter control (t = −1.9890; *p*-value = 0.0528), communication (t = −0.4981; *p*-value = 0.6206), and social cognition (t = −0.5288; *p*-value = 0.5985). The SF-36 questionnaire (Figure 4c) revealed significant differences between the initial and follow-up evaluations in the domains of bodily pain (t = −3.6703; *p*-value = 0.0022), general health (t = −2.3381; *p*-value = 0.0281), mental health (t = −3.3018; *p*-value = 0.0030), physical functioning (t = −2.2575; *p*-value = 0.0339), role-emotional (t = −2.6363; *p*-value = 0.0161), social functioning (t = −3.1250; *p*-value = 0.0049), and vitality (t = −3.0302; *p*-value = 0.0059). The only item that did not exhibit a significant improvement was the role-physical (t = −1.8680; *p*-value = 0.0763).

**Figure 5:**
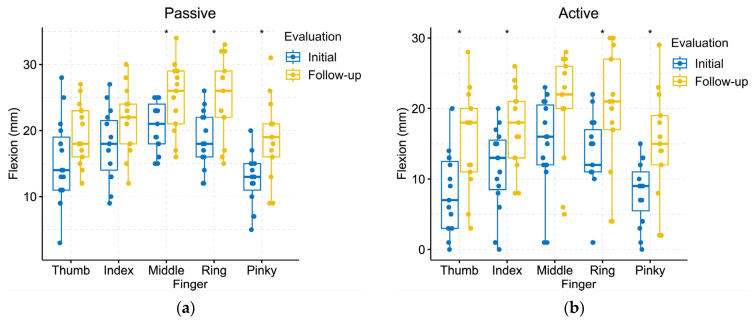
Goniometry measurements expressed in mm obtained using the HandTutor glove for the 13 participants during the initial and follow-up evaluations: (**a**) Passive range of motion for each finger; (**b**) Active range of motion for each finger. * indicates significant differences identified by a paired *t*-test at the *p* < 0.05 level.

Figure 5 illustrates the finger range of motion measured by the HandTutor glove during both the initial and follow-up evaluations. The passive flexion, measured in millimeters, exhibited an average increase of 60.4 ± 25.7%, while the active flexion showed an average increment of 28.7 ± 11.2%. Specifically, the passive flexion increased by 6.4 ± 0.8 mm, while active flexion recorded a mean increase of 28.7 ± 11.2 mm. Regarding passive range of motion (Figure 5a), significant differences were observed in the flexion of three fingers: middle (t = −2.4486, *p* = 0.0231), ring (t = −3.0195, *p* = 0.0066), and little (t = −2.9665, *p* = 0.0076). No significant differences were noted between the thumb (t = −2.0519, *p* = 0.0507) and index (t = −1.8067, *p* = 0.0826). Concerning active range of motion (Figure 5b), significant differences were found in the flexion (in mm) of four fingers: thumb (t = −2.9858, *p* = 0.0067), index (t = −2.6579, *p* = 0.0134), ring (t = −2.2641, *p* = 0.0343), and little (t = −2.8849, *p* = 0.0097), while no significant differences were observed in the middle (t = −1.9394, *p* = 0.0642).

All numerical results obtained from the clinical evaluation instruments and motion analysis conducted at the initial and follow-up evaluations are available in Table S1.

## 4. Discussion

In this study, thirteen individuals who had experienced musculoskeletal complications following severe COVID-19 infection participated in a six-week program of physical robotic-assisted hand rehabilitation. The study outcomes reveal significant improvements across various domains of physical function and health-related quality of life. However, it is important to critically analyze these results and consider potential causes of the observed enhancements.

Significant improvements were observed in the Barthel Index (BI), specifically in the self-care sub-index. This suggests that hand rehabilitation has had a positive impact on patients’ ability to perform essential daily activities by themselves. However, the mobility index did not show a significant improvement as it does not specifically target the dexterity and precision of the upper limb or hand mobility but rather evaluates overall mobility skills, which mainly depend on lower extremities’ motor function. In fact, it focuses on assessing a person’s ability to move and perform basic activities such as getting out of bed, sitting down, standing up, walking, and climbing stairs. Consequently, hand rehabilitation, while beneficial for self-care tasks which require the fine mobility of the upper limb, especially the hand, has not had a significant effect on overall mobility. Moreover, although all patients, except for one, exhibited improvements in the overall BI index, no statistically significant differences were observed. Notably, the only patient who did not show improvement, maintaining the same index, was the one with total dependence.

Regarding the Functional Independence Measure (FIM), significant improvement was observed in several items, including locomotion, self-care, and transfers. This is encouraging as it indicates that hand rehabilitation has positively contributed to patients’ functional independence in these areas. In both the BI and the FIM tests, the self-care area has improved. On the other hand, the reason why significant differences were found in locomotion and transfers subscales in the FIM but not in the mobility index of the BI may be attributed to the greater sensitivity and detail in the assessment of mobility in the FIM [37]. The FIM breaks down activities into more specific subcomponents, allowing for the detection of subtler improvements in mobility, including those related to the fine and precise mobility of the upper limb. In contrast, the mobility index of the BI may be less sensitive to detect improvements in specific areas of mobility, as it focuses on general mobility activities and may overlook smaller or specific changes in upper limb function.

Nevertheless, it is worth noting that significant improvements were not found in the domains of social cognition, communication, and sphincter control. This lack of improvement can be attributed to the fact that these aspects are not inherently connected to hand motor function. In the communication domain, none of the participants used sign language, which involves the use of hands for communication. These three aspects received the highest scores in the pre-assessment (communication 6.31 ± 1.19, social cognition 6.59 ± 0.94, and sphincter control 5.38 ± 2.10 using the 7-point Likert scale), indicating a low level of issues in these areas. Furthermore, addressing these domains may necessitate additional therapeutic interventions. The total FIM score also did not show significant improvements, indicating that although there were enhancements in specific domains, the overall recovery of independence did not demonstrate comprehensive improvement. This complexity could arise from the diverse health issues faced by patients who had been in the ICU.

In terms of the SF-36 test, significant improvements were observed in several quality of life-related items, such as bodily pain, general health, mental health, physical functioning, role-emotional, social functioning, and vitality. This could be attributed to improved hand function, as the ability to perform daily tasks and participate in social and physical activities may have improved. The only item that did not improve significantly is the role-physical since it evaluates specific activities related to social and occupational roles that require adequate physical functioning, and improvements in fine upper limb mobility may not have a direct impact on these activities. Additionally, it may suggest that other factors such as generalized muscle weakness or fatigue still limit patients’ ability to perform certain physical roles.

The goniometry results performed using the HandTutor provided data related to the range of motion, specifically, passive flexion and active flexion. This instrument has been employed in previous studies for assessing hand motor function [36,38,39]. Although active kinematics are more accurate for evaluating the degree of motor recovery, it has been demonstrated that even passive kinematics can provide an overview of the biomechanical behavior of the studied limb [40]. Therefore, according to the presented results, it can be concluded that the range of motion of the fingers of the patients significantly increased after the rehabilitation with the RobHand exoskeleton.

A prior investigation evaluated functional outcomes using robotic therapy with COVID-19 patients. Tay et al. [27] conducted gait robotic rehabilitation using ANDAGO in a cohort of 13 patients, averaging 65 years of age, with an average hospitalization period of 38 days, including 7 days spent in the ICU. Their findings demonstrated notable enhancements in the FIM total score from admission to discharge. Cevei et al. [26] conducted a clinical trial with six elderly patients (all over 80 years old) using LOKOMAT for gait robotic rehabilitation over a span of 4 weeks. With an average hospital stay of 17 days and no ICU admissions, significant improvements were observed in the FIM total score but not in the BI, attributing the results to the limited sample size and the advanced age of the participants. Zaskasda et al. [28] conducted a comprehensive randomized controlled trial with 30 participants, implementing robotic therapy with LUNA EMG in addition to other physical exercises for the intervention group, while the control group received only physical therapy exercises for movement coordination and balance. They observed significant increases in both FIM and BI scores. Our 4-week study comprised 13 patients with an average age of 60.2 years, the youngest among the aforementioned studies. Importantly, the patients in this clinical trial experienced prolonged hospitalizations (mean hospital stay of 52 days and ICU stay of 38 days on average), indicating a more severe COVID-19 course due to stricter admission criteria. Distinguishing itself from previous investigations, our rehabilitation approach exclusively targeted hand robotic-assisted rehabilitation, yielding notable improvements in hand motor function-related items within both the FIM and BI scales.

This study has some limitations. One notable limitation is the relatively small sample size (*n* = 13). A larger and more diverse sample would provide a more comprehensive understanding of the subject under investigation. Secondly, the absence of a control group in the study design limits the ability to make direct comparisons and draw conclusions about the causative effects of rehabilitation. Additionally, as the participants’ pre-COVID-19 states are unknown, it remains challenging to ascertain whether the rehabilitation achieved optimal levels or merely induced improvements. Furthermore, without the knowledge of their pre-COVID-19 conditions, it is challenging to assess the extent of their initial impairments. While Karasu et al. [41] noted significant enhancements in muscle strength, physical performance, and musculoskeletal symptoms in COVID-19 patients over time, these physical improvements did not return to normal levels. As a result, they concluded that post-COVID-19 rehabilitation programs are essential.

Additionally, care should be exercised when generalizing the findings to all COVID-19 patients. The study’s participants were specifically those eligible for patients who had been previously hospitalized, potentially excluding patients with milder symptoms who were discharged home directly. The study only assessed patients at two time points. It would be interesting to consider a longer-term follow-up, such as 3–6 monthly intervals, to evaluate patients’ reintegration and function in the community, providing a more comprehensive view of the rehabilitation outcomes.

Lastly, the study assesses the subject’s functional status using various measures like the Barthel Index, FIM, and SF-36. However, it only includes an evaluation of the upper limb, specifically the hand. Consequently, the study lacks data related to the lower limb or other body regions, making it incomplete in drawing comprehensive conclusions about the patient’s overall functional status and the rehabilitation outcomes.

The findings of this study hold valuable implications for healthcare professionals involved in rehabilitating severe COVID-19 patients. Understanding the effectiveness of hand robotic rehabilitation can guide the development of tailored rehabilitation programs, which have been underscored to be essential for mitigating fatigue and enhancing functional outcomes in COVID-19 patients [14]. Additionally, future studies can build upon these findings by exploring different modalities of robotic rehabilitation and their long-term effects. Collaborative efforts between researchers, clinicians, and technology developers can further advance robotic-assisted rehabilitation, benefiting not only COVID-19 patients but also others with similar needs, such as stroke patients or individuals requiring prehabilitation [42]. Additionally, these findings can help prepare for future pandemics by laying the groundwork for high-quality robotic rehabilitation programs that alleviate the strain on healthcare personnel while ensuring optimal patient recovery, even in resource-constrained settings.

## 5. Conclusions

This study provides valuable insights into the implementation of robotic rehabilitation for individuals recovering from upper limb impairments post-COVID-19. The utilization of a hand rehabilitation exoskeleton yielded positive outcomes, contributing to the physical and psychological well-being of patients. These favorable results underscore the significance of such interventions, especially amid the strain on healthcare systems during the heightened challenges of the COVID-19 pandemic. Robotic systems like these serve as crucial tools, relieving the burden on healthcare professionals by allowing them to allocate less time to rehabilitation while ensuring patients receive necessary medical attention and physical therapy. Given the multifaceted nature of recovery for severe COVID-19 patients, it is imperative to carefully consider various factors, including the role of robotic rehabilitation in the broader context of health and mobility recovery. This consideration is crucial for interpreting results and formulating effective treatment strategies within the unique challenges posed by the global pandemic’s impact on healthcare systems.

## Figures and Tables

**Table 1:** Assessment of domains of the International Classification of Functioning, Disability and Health.

Domains	Components	Outcome Measure ^1^
Body functions and structures	Genitourinary	FIM, sphincter control
	Mental functions	SF-36, mental health SF-36, vitality
	Sensory functions and pain	SF-36, bodily pain SF-36, general health SF-36, physical functioning SF-36, role-physical
	Neuromusculoskeletal and movement-related functions	Motion analysis, goniometry
Activities and participation	Communication	FIM, communication
	Mobility	BI, mobility FIM, locomotion FIM, transfers
	Personal care	BI, health care FIM, self-care
	Community, social, and civic life	FIM, social cognition SF-36, social functioning
	Emotional role	SF-36, role-emotional
	Domestic life	Interview, living arrangements
Environmental factors	Products and technology	Interview, electronic devices
Personal factor	Level of education	Interview

^1^ FIM: Functional Independence Measure; BI: Barthel Index; SF-36: SF-36 Health Questionnaire.

**Table 2:** Overview of demographic characteristics, clinical information, living situation, educational background, and the use of electronic devices in the study population.

Demographic Characteristics	
Age (years)	60.2 ± 16.0 [range 41–83]
Gender	Male: 8 (61.5%); Female: 5 (38.5%)
**Clinical information**	
Upper limb impairments	Left: 5 (38.5%); Right: 8 (61.5%)
Infection to study initiation time (months)	3.8 ± 2.1 [range 1–8.5]
Hospitalization duration (days)	52.2 ± 40.1 [range 10–109]
Ward stay duration (days)	38.7 ± 27.0 [range 10–78]
ICU admission required	Yes: 5 (38.5%); No: 8 (61.5%)
ICU stay duration (days)	27.0 ± 11.2 [range 7–33]
**Other clinical complications**	
Stroke	Yes: 5 (38.5%); No: 8 (61.5%)
Stroke to study initialization time (months)	2.8 ± 2.3 [range 1–7]
Lymphoma	Yes: 1 (7.7%); No: 12 (92.3%)
Lymphoma to study initiation time (months)	12
**Living situation**	
Living place	Residential/hospital facilities: 3; House: 10
Living arrangement in house	Family member/partner: 8; Alone: 2
**Educational background**	
University: 4; Primary: 4; Secondary: 1; High school: 2; Professional training: 2	
**Use of electronic devices**	
Mobile phone	Rarely: 4; Occasional: 2; Frequent: 7
Video game console	Never: 11; Occasional: 2
Computers	Never: 7; Occasional: 1; Frequent: 5

**Table 3:** **The** level of dependence of the participants at the initial and follow-up evaluation based on the Barthel Index.

Barthel Index	Level of Dependence	Number of Participants	
		Initial Evaluation	Follow-Up
0–20	Total dependence	2	1
21–60	Severe dependence	6	1
61–90	Moderate dependence	4	5
91–99	Slight dependence	1	1
100	Independence	0	5

## Data Availability

The data presented in this study are available on request from the corresponding author.

## Supplementary Materials

The following supporting information can be downloaded at: https://www.mdpi.com/article/10.3390/jcm13061543/s1, Table S1: Results from the Barthel Index, the Manual Independence Function, and SF-36 tests, and the active and passive joint range (in mm) measured using HandTutor. Initial and follow-up evaluation data with confidence intervals, *p*-values, and significance.
