# Supplementary material for: Tackling Post-COVID-19 Rehabilitation Challenges: A Pilot Clinical Trial Investigating the Role of Robotic-Assisted Hand Rehabilitation
Source: J Clin Med. 2024 Mar 7;13(6):1543. doi: 10.3390/jcm13061543 (PMC10971451; doi:10.3390/jcm13061543)
Supplement: Supplementary file 1 [file jcm-13-01543-s001.zip › jcm-2867682-supplementary.pdf]

Table S1. Results from the Barthel Index, the Manual Independence Function and SF-36 tests, and the active and passive joint range (in mm) measured using HandTutor. Initial and follow-up evaluation data with confidence intervals, p-values, and significance.

	**Initial evaluation Mean±SD [Q1, Q3]**	**Follow-up Mean±SD [Q1, Q3]**	**Confidence interval**	**p-value**	**Sig.**
		**BARTHEL INDEX (BI)**			
Self-care index	31.92±12.02 [21, 42]	45.54±12.51 [44, 53]	[-23.05, -3.68]	0,0093	*
Mobility index	24.30±17.28 [10, 37]	33.15±15.24 [25, 47]	[-22.05, 4.36]	0.1793	
Total score	56.23±28.75 [31, 83]	78.69±27,07 [69, 100]	[-45.07, 0.15]	0.0514	
		**Functional Independence Measure (FIM)**			
Communication	6.31±1.19 [5.5, 7]	6.46±1.03 [7, 7]	[-0.77, 0.47]	0.6206	
Locomotion	3.77±2.23 [1.25, 5]	5.12±2.16 [4, 7]	[-2.57, -0.12]	0.0318	*
Self-Care	4.22±1.99 [3, 6.25]	5.89±1.74 [4, 7]	[-2.27, -1.07]	0.0000	*
Social cognition	6.59±0.94 [7, 7]	6.69±0.77 [7, 7]	[-0.49, 0.28]	0.5985	
Sphincter control	5.38±2.10 [3, 7]	6.38±1.47 [7, 7]	[-2.01, 0.01]	0.0528	
Transfers	4.91±1.47 [2, 7]	6.00±1.37 [4, 7]	[-2.52, -0.45]	0.0054	*
Total	87.62±26.34 [62, 112]	107.15±24.87 [102, 124]	[-40.28, 1.20]	0.0636	
		**SF-36**			
Bodily Pain (BP)	55.19±34.32 [22.5, 87.5]	92.50±12.87 [90, 100]	[-58.94, -15.68]	0.0022	*
General Health (GH)	37.31±24.63 [20, 45]	58.85±22.28 [45, 80]	[-40.56, -2.52]	0.0281	*
Mental Health (MH)	45.85±22.01 [36, 52]	74.46±22.18 [60, 96]	[-46.50, -10.73]	0.0030	*
Physical Functioning (PF)	22.31±30.04 [0, 25]	52.69±38.11 [15, 90]	[-58.24, -2.52]	0.0339	*
Role Emotional (RE)	10.26±28.50 [0, 0]	51.28±48.33 [0, 100]	[-73.55, -8.50]	0.0161	*
Role Physical (RP)	7.69±27.74 [0, 0]	34.62±43.94 [0, 75]	[-56.96, 3.12]	0.0763	
Social Functioning (SF)	25.00±28.41 [0, 37.5]	66.35±38.32 [37.5, 100]	[-68.78, -13.92]	0.0049	*
Vitality (VT)	40.77±21.30 [30, 55]	68.08±24.54 [55, 85]	[-45.93, -8.69]	0.0059	*
		**Active flexion (mm)**			
Thumb	7.93±5.69 [3.00, 12.50	15.38±7.27 [11, 20]	[-12.62, -2.28]	0.0067	*
Index	11.47±5.90 [8.50, 15.50	17.38±5.85 [13, 21]	[-10.50, -1.34]	0.0134	*
Middle	14.80±6.82 [12.00, 20.50	20.15±7.67 [20, 26]	[-11.05, 0.34]	0.0642	
Ring	12.93±6.11 [12.93, 20.50]	19.54±8.85 [17, 27]	[-12.67, -0.54]	0.0343	*
Little	7.87±4.39 [5.50, 11.00]	14.92±7.82 [12, 19]	[-12.19, -1.92]	0.0097	*
		**Passive flexion (mm)**			
Thumb	15.07±6.55 [11.0, 19.0]	19.46±4.74 [16.0, 23.0]	[-8.80, 0.01]	0.0507	
Index	17.80±5.25 [14.0, 21.5]	21.38±5.22 [18.0, 24.0]	[-7.67, 0.50]	0.0826	
Middle	20.67±3.75 [18.0, 24.0]	25.00±5.34 [21.0, 29.0]	[-8.01, -0.65]	0.0231	*
Ring	18.67±4.27 [16.0, 22.0]	24.85±6.22 [22.0, 29.0]	[-10.44, -1.92]	0.0066	*
Little	12.67±4.03 [11.0, 15.0]	18.69±6.29 [16.0, 21.0]	[-10.26, -1.79]	0.0077	*

* indicates significant differences identified by a paired t-test at the p<0.05 level.
